# Delayed Diagnosis of Pharyngeal Perforation following Exploding Tyre Blast Barotrauma

**DOI:** 10.1155/2014/382495

**Published:** 2014-11-26

**Authors:** Samantha M. Field, Joseph G. Manjaly, S. Krishan Ramdoo, Huw A. S. Jones, Taran S. Tatla

**Affiliations:** Department of Otolaryngology-Head and Neck Surgery, Northwick Park Hospital, Watford Road, Harrow, Middlesex HA1 3UJ, UK

## Abstract

*Introduction*. Pharyngoesophageal perforation secondary to barotrauma is a rare phenomenon that can have serious complications if identified late. It is challenging to detect due to nonspecific symptoms. We present a case in which detection proved difficult leading to delayed diagnosis.* Case Report*. A 27-year-old mechanic presented with haemoptysis, dysphonia, and odynophagia after a car tyre exploded in his face. Flexible nasoendoscopy (FNE) revealed blood in the pharynx, thought to represent mucosal haemorrhage. Initial treatment consisted of IV dexamethasone and antibiotics. After 3 days, odynophagia persisted prompting a CT scan. This revealed a defect in the posterior hypopharynx and surgical emphysema in the deep neck tissues. Contrast swallow confirmed posterior hypopharyngeal leak. NG feeding was commenced until repeated contrast swallow confirmed resolution of the defect.* Discussion*. Prompt nonsurgical management of pharyngoesophageal perforation has good outcomes but untreated perforation can have serious complications. FNE should be performed routinely, but only a contrast swallow can diagnose a functional perforation. Clinicians should have a high index of clinical suspicion when patients present with barotrauma and odynophagia. Patients should be kept nil by mouth until perforation has been excluded.* Conclusion*. When faced with cases of facial barotrauma, clinicians should have a low threshold for further imaging to exclude pharyngoesophageal perforation.

## 1. Introduction

Pharyngoesophageal perforation secondary to barotrauma is a rare phenomenon that can be difficult to detect in the acute setting. It can present with nonspecific symptoms and thus prove difficult to detect on initial presentation. We present a case of pharyngeal perforation secondary to barotrauma which followed this rule.

## 2. Case Report

A previously healthy 27-year-old Polish mechanic presented to A & E after an overinflated car tyre exploded in his face. At the time he complained of some respiratory difficulty, a small amount of haemoptysis, and dysphonia. Whilst in the emergency department, ophthalmologists removed bilateral corneal foreign bodies and the maxillofacial surgeons identified a chipped L2 buccal crown. An ear, nose, and throat examination of the neck revealed no evidence of surgical emphysema, spinal tenderness, or any obvious external deformity, and there were no symptoms of chest pain or respiratory compromise. Flexible Nasoendoscopy (FNE) revealed blood in posterior pharynx and around the vocal cords but no areas of active bleeding. The blood was initially felt to be secondary to mucosal haemorrhage. He was admitted to the ENT ward for observation with prophylactic IV coamoxiclav and IV dexamethasone 8 mg BD. The following day he was commenced on a soft diet to aid his swallow and kept in for observation as he complained of odynophagia.

**Figure 1:**
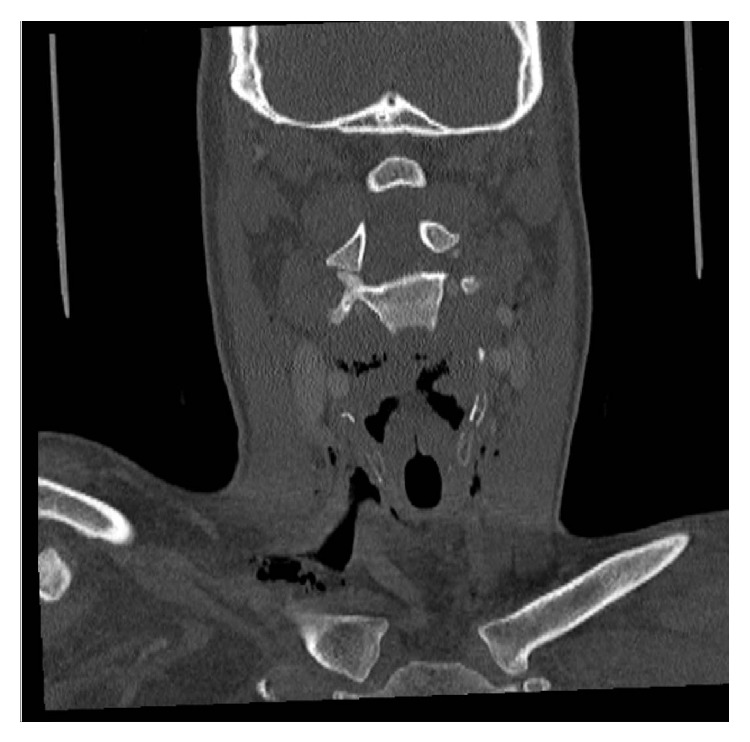
Computed tomography showing extensive surgical emphysema in the deep neck tissues.

After 48 hours, the odynophagia persisted, and there was a suspicion of some mild palpable emphysema over the thyroid cartilage. A repeat FNE was unremarkable with no further bleeding identified. A CT neck was performed (Figure 1) which revealed a defect in the posterior wall of the hypopharynx on the left side measuring 7 mm in width and 30 mm craniocaudally. In addition there was extensive surgical emphysema in the deep neck tissues, with gas tracking superiorly in the retropharyngeal space up to the level of the nasopharynx, into the left parapharyngeal space, the masseteric space, and posteriorly between the left carotid and parotid. It was also seen inferiorly in the retropharyngeal space, extending inferiorly and anteriorly into the superior mediastinum including around the thoracic outlet structures and into the supraclavicular regions.

**Figure 2:**
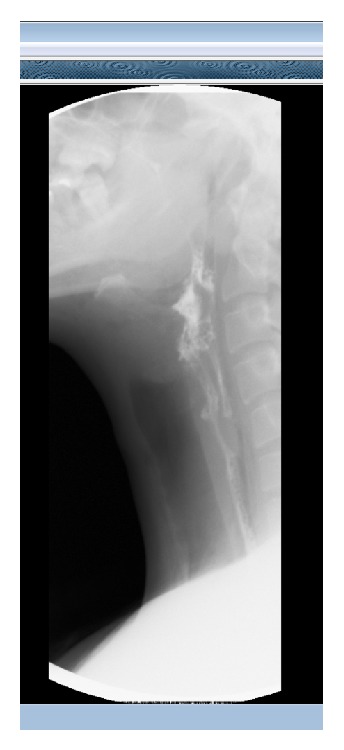
Water soluble contrast swallow showing a posterior hypopharyngeal leak with liquid tracking down the prevertebral space.

A water soluble contrast swallow confirmed there was a posterior hypopharyngeal leak and showed liquid tracking down the prevertebral space (Figure 2). He was therefore commenced on NG feeding for 1 week. A repeat contrast swallow at this point confirmed resolution of the defect and he was discharged home safely, symptom free.

## 3. Discussion

Pharyngoesophageal perforation secondary to barotrauma historically has been mainly due to exploding drink bottles, often when the patient tries to open them with their teeth, but there have been less than 30 cases of this recorded in the English literature [1, 2]. Vehicle or bicycle tyre explosion causing pharyngoesophageal barotrauma is an uncommon mechanism of insult. It has been documented following children biting tyres [3, 4] and there has been one reported case of tyre explosion by this mechanism in an adult [5]. In this case an oesophageal rupture was picked up after a CT chest was performed to investigate pleural effusions.

Early nonsurgical management of pharyngoesophageal perforation has been shown to have good outcomes if implemented early [6]. However, a delay in diagnosis until after the patient has eaten can lead to salivary leak into the neck tissues causing serious complications such as deep neck abscess or mediastinitis. For this reason a perforation with a delayed diagnosis has been shown to have high rates of mortality (16–75%) and morbidity (35%–66%) [7]. It is therefore essential to have a high index of clinical suspicion when a patient presents with barotrauma, odynophagia, and blood in pharynx. Other injuries, such as ocular damage, should not distract from investigation into possible perforation.

The first line method for detecting perforation is orally ingested radiopaque contrast scanning. Flexible Nasoendoscopy should be performed as routine to identify any defects in the mucosa as well as injuries to other structures. However, it is worth noting that, in this case, repeated endoscopic evaluation did not reveal any defect and the patient was thus allowed to eat and drink freely. Only after he complained of persistent pain on swallowing which limited his oral intake was a CT scan performed. However, although a CT scan can demonstrate surgical emphysema and mucosal defects, only contrast scanning can show a functional perforation. It has long been established that barium is not an irritant to the mediastinum in the same way that it is to the peritoneum, and therefore a barium swallow is considered a safe method for investigating pharyngeal perforation [8]. However, a study comparing barium and aqueous based contrast mediums observed a slight benefit over using aqueous based mediums due to a lower viscosity, which increases its sensitivity for delineating finer tracts [9].

## 4. Conclusion

Pharyngeal perforation is a potentially serious condition that should be identified as early as possible for the best outcome. Unfortunately it often presents with nonspecific symptoms that can be overlooked when there are other, more obvious injuries. For this reason, in patients presenting with barotrauma there should be a low threshold for investigation with contrast swallow. All patients presenting with barotrauma should be kept nil by mouth until perforation has been excluded.
